# Supplementary material for: GRIDGENE: Guided Region Identification based on Density of GENEs—a transcript density-based approach to characterize tissues by spatial transcriptomics
Source: Bioinform Adv. 2026 Aug 25;6(1):vbag249. doi: 10.1093/bioadv/vbag249 (PMC13641078; doi:10.1093/bioadv/vbag249)
Supplement: Supplementary file 1 [file vbag249_supplementary_data.docx]

**Supplementary information**

Supplementary figure 1 – Influence of kernel size on mask generation. Example from a Xenium dataset illustrating how varying the kernel size affects the shape and granularity of cancer masks. The stroma mask remains constant, while the cancer mask is computed using kernel sizes of 10 µm, 20 µm, and 30 µm for the convolutional sum. Larger kernel sizes produce smoother and more coarsened contours, while smaller kernels result in more fine-grained and detailed masks.

**Supplementary Figure 2 – Quantitative validation of GRIDGENE compartment masks against cell segmentation in CosMx data.** (A) Cell-level comparison between major cell population and GRIDGENE mask assignment for each FOV. Left: cells coloured by their phenotype, derived from cell segmentation and clustering. Middle: cells coloured by the GRIDGENE mask they predominantly overlap with, based on pixel-level overlap. Right: agreement map, where red and blue cells are correctly assigned and yellow cells indicate a discordant assignment between cell phenotype and GRIDGENE mask. (B) Quantification of cell phenotype distribution across GRIDGENE compartments. Left: stacked bar plot showing the proportion of each cell type assigned to tumour or stroma regions, aggregated across all FOVs. Middle and right: heatmaps showing the percentage of each cell phenotype assigned to the tumour (middle) and stroma (right) masks per FOV. Overall concordance between GRIDGENE mask assignment and cell phenotype was 96.0% across all FOVs.

**Supplementary Figure 3 – Quantitative validation of GRIDGENE compartment masks against cell segmentation in Xenium data.** (A) Cell-level comparison between major cell populations and GRIDGENE mask assignment for each FOV. Left: cells coloured by their phenotype. Middle: cells coloured by the GRIDGENE mask they predominantly overlap with, based on pixel-level overlap. Right: agreement map, where red and blue cells are correctly assigned and yellow cells indicate a discordant assignment between cell phenotype and GRIDGENE mask. (C) Quantification of cell phenotype distribution across GRIDGENE compartments. Left: stacked bar plot showing the proportion of each cell type assigned to tumour or stroma regions, aggregated across all FOVs. Middle and right: heatmaps showing the percentage of each cell phenotype assigned to the tumour (middle) and stroma (right) masks per FOV. Overall concordance between GRIDGENE mask assignment and cell phenotype was 97.8% across all FOVs.

**Supplementary Figure 4 – Quantitative validation of GRIDGENE compartment masks against reference annotations in CosMx data.** (A): Comparison of reference masks and GRIDGENE-generated masks for three representative FOVs. From left to right: immunofluorescence (IF) reference image; manually annotated reference mask; GRIDGENE-predicted mask; agreement map where red indicates correctly predicted tumour, blue indicates correctly predicted stroma, and yellow indicates disagreement between the two annotations. (B): segmentation performance metrics (Dice, IoU, Precision, Recall, F1) for tumour and stroma compartments across the three FOVs, shown as mean ± standard deviation with individual FOV values overlaid.

s

**Supplementary Figure 5 – GRIDGENE-identified γδ T cell regions in Xenium data validated against IMC.** Zoomed in IMC composite images for three representative ROIs, with in the left panel TCRγδ (blue) and DNA (white) and in the middle panel TCRγδ (blue) and Keratin (white). In the right panel: corresponding GRIDGENE-generated masks, where grey indicates the tumour compartment and blue dots indicate putative γδ T cell regions identified based on co-localisation of TRGC and TRDC transcripts. Arrows indicate examples of overlapping TCRγδ signal in IMC with GRIDGENE identified γδ T cell regions.

**Supplementary figure 6 – Alternative strategies for spatial masking using GRIDGENE.** (A) Xenium DAPI references from three CRC samples. (B) Cancer and stroma regions were masked using a self-organizing map (SOM)–based approach, where 10×10 pixel grids were clustered using a SOM with two units. Although unsupervised, this method appears to separate stroma and cancer compartments. (C) Heatmap showing genes differentially expressed between the two SOM-derived clusters, supporting their identification as stroma and cancer. (D) Alternative cancer/stroma masks were generated by calculating local point density using a KD-Tree with a 10 µm radius, then deriving contours based on neighbourhood counts. (E) Putative γδ T cells were identified by locating TRGC and TRDC transcripts that are ≤3 µm apart. Circular regions were drawn around these pairs and discarded if TRAC or TRBC signals were more abundant. These regions can be overlaid with cell segmentation data to identify γδ T cells or used directly as spatial regions of interest.

**Supplementary Figure 7 – Runtime and memory benchmarking of GRIDGENE region detection methods.** Runtime in seconds for CosMx (A) and Xenium (B) for the Convolutional, KD-tree, and SOM methods at increasing data scales (1, 5, and 10 samples processed sequentially). CosMx FOVs are 500×500 µm regions imaged at 4245×4245 pixels (~0.12 µm/pixel) profiled for 999 genes; Xenium cores are 1500 µm diameter regions at 1500×1500 pixels (1 µm/pixel) profiled for 480 genes. For Xenium, an additional data point indicates performance on approximately one quarter of a full Xenium slide (~6.9M transcripts). Numbers in parentheses on the x-axis indicate total transcripts processed at each scale. (C, D) Peak memory usage (GB) for the same conditions. For the SOM method, all samples at a given scale are processed jointly; for Convolutional and KD-tree, each sample is processed independently. Benchmarks were performed on a Linux x86_64 system with 8 CPU cores and 62.6 GB RAM.

**Supplementary Table 1** – **GRIDGENE benchmark summary.** Runtime and peak memory usage for the Convolutional, KD-tree, and SOM region detection methods across CosMx and Xenium platforms at increasing data scales.

| **Platform** | **Method** | **Scale** | **Transcripts (M)** | **Runtime mean (s)** | **Runtime std (s)** | **Memory mean (GB)** | **Memory std (GB)** |
| --- | --- | --- | --- | --- | --- | --- | --- |
| CosMx | Convolutional | 1 core | 1.84 | 31.7 |  | 40.84 |  |
| CosMx | Convolutional | 5 cores | 5.44 | 28.96 | 1.99 | 40.65 | 0.2 |
| CosMx | Convolutional | 10 cores | 10.53 | 29.03 | 2.27 | 40.63 | 0.21 |
| CosMx | KD-tree | 1 core | 1.84 | 83.29 |  | 33.4 |  |
| CosMx | KD-tree | 5 cores | 5.44 | 52.5 | 22.6 | 15.92 | 11.76 |
| CosMx | KD-tree | 10 cores | 10.53 | 50.78 | 22.54 | 15.38 | 13.12 |
| CosMx | SOM | 1 core | 1.84 | 2.82 |  | 1.01 |  |
| CosMx | SOM | 5 cores | 5.44 | 7.1 |  | 1.01 |  |
| CosMx | SOM | 10 cores | 10.53 | 13.5 |  | 1.46 |  |
| Xenium | Convolutional | 1 core | 2.49 | 16.15 |  | 3.04 |  |
| Xenium | Convolutional | 5 cores | 17.24 | 22.38 | 6.02 | 2.97 | 0.22 |
| Xenium | Convolutional | 10 cores | 31 | 19.95 | 5.51 | 2.88 | 0.28 |
| Xenium | Convolutional | Full slide (1/4 Xenium) | 6.93 | 164.03 |  | 38.91 |  |
| Xenium | KD-tree | 1 core | 2.49 | 44.93 |  | 10.26 |  |
| Xenium | KD-tree | 5 core | 17.24 | 64.14 | 23.56 | 17.49 | 8.77 |
| Xenium | KD-tree | 10 cores | 31 | 56.25 | 21.41 | 14.95 | 7.89 |
| Xenium | KD-tree | Full slide (1/4 Xenium) | 6.93 | 244.6 |  | 32.22 |  |
| Xenium | SOM | 1 core | 2.49 | 9.66 |  | 0.78 |  |
| Xenium | SOM | 5 cores | 17.24 | 69.89 |  | 2.04 |  |
| Xenium | SOM | 10 cores | 31 | 125.93 |  | 3.67 |  |
| Xenium | SOM | Full slide (1/4 Xenium) | 6.93 | 117.79 |  | 22.05 |  |
